# Relationship between Thyroid Function and Kidney Function in Patients with Type 2 Diabetes

**DOI:** 10.1155/2018/1871530

**Published:** 2018-11-14

**Authors:** Ying Zhang, Yang Wang, Xiao Jun Tao, Qian Li, Feng Fei Li, Kok Onn Lee, Dong Mei Li, Jian Hua Ma

**Affiliations:** ^1^Department of endocrinology, Nanjing First Hospital, Nanjing Medical University, Nanjing, China; ^2^Department of endocrinology, Nanjing Pukou Central Hospital, Nanjing, China; ^3^Department of Medicine, National University of Singapore, Singapore, Singapore

## Abstract

**Purpose:**

To determine if the TSH is related to estimated glomerular filtration rate (eGFR) in T2D patients without overt thyroid dysfunction.

**Methods:**

A cohort study of 5936 T2D patients was assessed for thyroid and kidney functions, in whom 248 with subclinical hyperthyroidism and 362 with subclinical hypothyroidism. Serum creatinine and 24-hour urine albumin excretion (UAE) were collected. Chronic kidney disease (CKD) was defined as eGFR < 60 ml/min/1.73 m^2^.

**Results:**

Compared with euthyroid subjects, the patients with subclinical hypothyroidism had lower eGFR (82.7 ± 22.4 vs. 90.5 ± 22.4 ml/min/1.73 m^2^, *p* < 0.01), higher UAE (114 ± 278 vs. 88 ± 229 mg/24 h, *p* < 0.05), and high incidence of CKD (16.0% vs. 10.1%, *p* < 0.05). The participants with a TSH level between 0.55 and 3.0 *μ*IU/ml had a higher eGFR (91.4 ± 22.2 ml/min/1.73 m^2^) and a lower prevalence of CKD (9.5%) than those with higher TSH (3.01–4.78 *μ*IU/ml, 85.6 ± 22.7 ml/min/1.73 m^2^, *p* < 0.01 and 13.1%, *p* < 0.01). Linear logistic regression analysis showed that the eGFR was significantly negatively associated with TSH (OR: 0.519, 95% CI: 0.291–0.927, *p* < 0.05), after adjustment of confounders.

**Conclusion:**

High TSH was independently associated with decreased eGFR in type 2 diabetes patients without overt thyroid dysfunction. Our findings indicate that doctors who treat T2D patients should routinely measure the thyroid function.

## 1. Introduction

Many patients with T2D develop chronic kidney disease, which is defined based on glomerular filtration rate < 60 ml/min/1.73 m^2^ and/or the presence of albuminuria, as defined in the recommendations of the 2012 KDIGO practice guideline [1]. The kidney functions in patients with T2D were influence by many situations and medicine, such as hypertension [2], lipid profiles [3], glycemic variability [4], and the renin-angiotensin-aldosterone system blockade [5]. The development of significant CKD is associated with an increased risk of adverse cardiovascular outcomes and death in patients with T2D [6, 7].

Overt thyroid disease is relatively uncommon in T2D, and only a few studies investigated their relationship with kidney function in T2D. Some studies have shown that overt and subclinical hypothyroidisms were associated with reduced eGFR and increased risk of CKD [8–11] and that unresolved subclinical hypothyroidism was independently associated with the rate of renal function decline [12].

Despite overt thyroid disease being uncommon in type 2 diabetic patients, there is an increase in subclinical hypothyroidism in these patients when compared with healthy population [13], but it is not known if the change in eGFR and CKD in these patients with T2D is related to thyroid function, especially in patients within the normal range of TSH. We therefore assessed the association of thyroid function with eGFR, 24-hour urine albumin excretion (UAE), and CKD in a large cross-sectional population study of type 2 diabetic participants without overt thyroid dysfunction.

## 2. Materials and Methods

### 2.1. Study Population

A consecutive series of 6172 Chinese participants with type 2 diabetes who visited Nanjing First Hospital, Nanjing Medical University between January 2012 and December 2017 were enrolled in this retrospective observational study.

The study was approved by the Medical Ethics Committee of Nanjing First Hospital Affiliated to Nanjing Medical University, and written informed consent was obtained from all participants.

The patients who met the following criteria were excluded: patients with a previous known history of thyroid dysfunction (*N* = 133), thyroidectomy (*N* = 37), pituitary disease (*N* = 5), autoimmune renal diseases, renal artery stenosis, or end-stage renal disease under maintenance dialysis kidney disease (*N* = 11). We further excluded subjects who were younger than 18 years of age, women who were pregnant, subjects with urinary tract infection, or subjects who were receiving concurrent treatment with drugs that could affect the thyroid function (lithium, amiodarone, or iodine).

### 2.2. Clinical Measurements

Demographic and clinical data including comorbidities, the use of antidiabetic medications, and the use of angiotensin-converting enzyme inhibitors (ACEI) or angiotensin II receptor blockers (ARBs) were obtained from the medical records of these participants. Body mass index (BMI) was calculated based on body weight (in kilograms) divided by body height in meters squared (kg/m^2^).

Serum creatinine, fasting blood glucose (FBG), lipid profiles (total cholesterol, triglycerides, and high- and low-density lipoproteins) were analyzed by enzymatic assays (Olympus AU5400 autoanalyzer; Beckman Coulter, Japan). Glycated hemoglobin (HbA1c) was measured by high-performance liquid chromatography (Bio-Rad, USA). UAE was determined by chemiluminescence immunoassay (Siemens, Germany).

### 2.3. Thyroid Function

Thyroid function was assessed by measurement of TSH (reference range: 0.55–4.78 *μ*IU/ml, chemiluminescence immunoassay, Siemens, Germany) and FT4 (reference range: 11.5–22.7 pmol/l, chemiluminescence immunoassay, Siemens, Germany). Subclinical hyperthyroidism was defined as a TSH level < 0.55 mIU/l with a FT4 level within normal range. Subclinical hypothyroidism was defined as a TSH level > 4.78 mIU/l with a FT4 level within normal range. Euthyroid state was defined as a TSH level from 0.55 mIU/l to 4.78 mIU/l with a FT4 level within normal range.

### 2.4. UAE and CKD

Abnormal UAE was defined according to the criteria of the American Diabetes Association (UAE ≥ 30 mg/24 h). The definition of microalbuminuria was UAE 30–299 mg/24 h, and macroalbuminuria was UAE ≥ 300 mg/24 h. The Chronic Kidney Disease Epidemiology Collaboration (CKD-EPI) formula was harnessed to calculate the estimated glomerular filtration rate (eGFR), which was as follows: eGFR = *a* × (creatinine/*b*)*c* × 0.993 age, where *a* = 144 (female) or 141 (male), *b* = 0.7 (female) or 0.9 (male), and *c* = −0.329 (female with serum creatinine ≤ 0.7 mg/dl), −0.419 (male with serum creatinine ≤ 0.7 mg/dl), and− 1.209 (male and female with serum creatinine > 0.7 mg/dl). The presence of chronic kidney disease (CKD) was defined as having an eGFR < 60 ml/min/1.73 m^2^.

### 2.5. Statistical Analysis

SPSS version 23.0 for MAC was used in all analyses. The duration of diabetes was presented as mean ± standard error; the other descriptive statistics were presented as mean ± standard deviation and numbers (percentages) for categorical variables. The significance of differences between any two groups was assessed by *t*-test; one-way analysis of variance (ANOVA) was utilized for comparing three groups or more, and *χ*^2^-test was chosen to compare categorical variables. If significant differences were found using one-way ANOVA, a post hoc least significant difference test was used to define the effective factors. We used the linear logistic regression analysis to examine the association between eGFR and TSH, and binary logistic regression analysis was used to investigate the risk factors for CKD. We also used Pearson's test to analyze the correlation between eGFR levels and the clinical characteristics of the participants. A *p* value < 0.05 was considered statistically significant.

## 3. Results

### 3.1. Participant Characteristics

**Figure 1:**
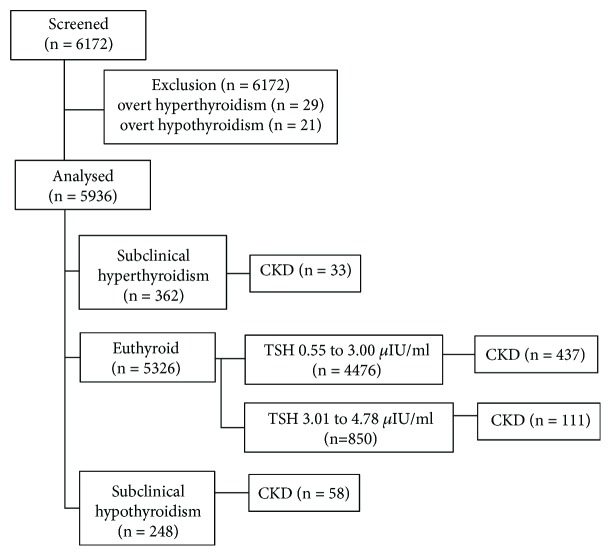
Flow chart of the study participant. CKD (chronic kidney disease) is defined as having an eGFR < 60 ml/min/1.73 m^2^.

Thyroid function testing found 29 patients with overt hyperthyroidism and 21 overt hypothyroidism; they were excluded from the analysis. A final total of 5936 patients with T2D were included into this study (Table 1 and Figure 1). These patients had a mean diabetic duration of 8.2 ± 6.8 years, with a mean HbA1c level of 9.2 ± 2.6%. Among these T2D patients, 1956 received insulin (either alone or in combination with sulfonylurea, metformin, or acarbose) and 1926 patients received sulfonylurea (either alone or in combination with metformin or acarbose). Metformin, which has been reported to lower TSH [14, 15], was used in 1127 subjects. Hypertension was present in 3547 (59.8%) subjects, and 1614 (45.5%) subjects were on ACEI or ARB.

### 3.2. UAE and CKD

Among the 5936 patients, 629 (10.6%) had evidence of CKD with an eGFR of <60 ml/min/1.73 m^2^, while 2087 (35.2%) had significant UAE, 1610 (27.1%) had microalbuminuria, and 477 (8.0%) had macroalbuminuria.

**Figure 2:**
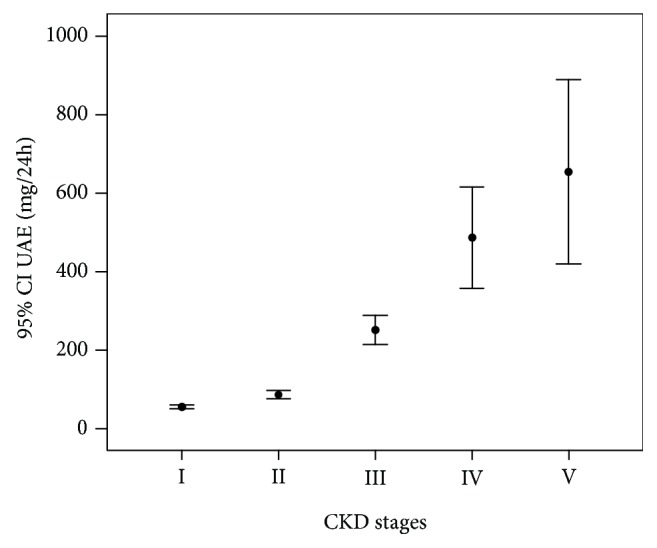
The mean and 95% CI of UAE in 5936 participants in type 2 diabetes without overt thyroid dysfunction according to different CKD stages.

The UAE levels increased with the CKD stages (all *p* < 0.01, Figure 2). The prevalence of DN, microalbuminuria, and macroalbuminuria also increased with the CKD stages (all *p* for trend < 0.01). Pearson correlation analysis showed that UAE was negatively correlated with eGFR (*r* = −0.0273, *p* < 0.01).

### 3.3. Thyroid Function

Among the 5936 patients, 5326 (89.7%) were euthyroid, 248 (4.2%) had subclinical hyperthyroidism, and 362 (6.1%) had subclinical hypothyroidism (Figure 1). Compared with euthyroid subjects, the patients with subclinical hypothyroidism were found more frequent female with the usage of metformin. The subclinical hypothyroid patients were also older, had higher BMI, longer duration of diabetes, higher HbA1c, and higher blood pressure. They also had lower eGFR, higher UAE, and more CKD (eGFR < 60 ml/1.73 m^2^). By contrast, the 248 patients with subclinical hyperthyroidism subjects did not have any of these significant changes (Table 1). No significant differences were found in the prevalence of DN, microalbuminuria, or macroalbuminuria among the three groups.

### 3.4. Renal Function and CKD Related to TSH Levels

**Figure 3:**
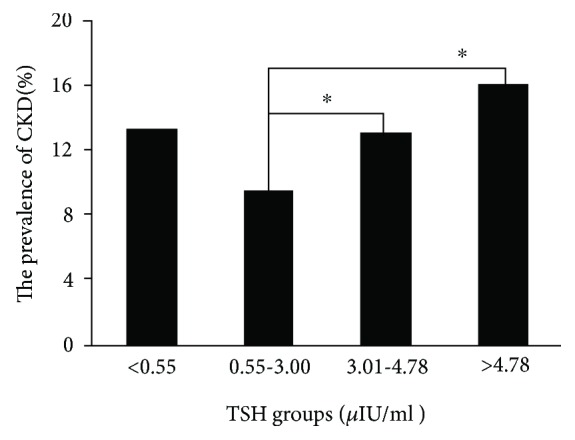
The prevalence of CKD in 5936 participants in type 2 diabetes without overt thyroid dysfunction according to TSH quartiles. ^∗^*p* < 0.05.

In 5326 participants with normal TSH, 4476 participants are with a TSH level from 0.55 to 3.00 *μ*IU/ml and 850 participants are with a TSH level between 3.01 and 4.78 *μ*IU/ml. Among 4476 with a TSH level from 0.55 to 3.00 *μ*IU/ml, 427 (9.5%) participants had CKD, lower than that in participants with a TSH level between 3.01 and 4.78 *μ*IU/ml (111 of 850, 13.1%, *p* < 0.02) and participants with subclinical hyperthyroidism (16.0%, *p* < 0.01, Figure 3).

**Figure 4:**
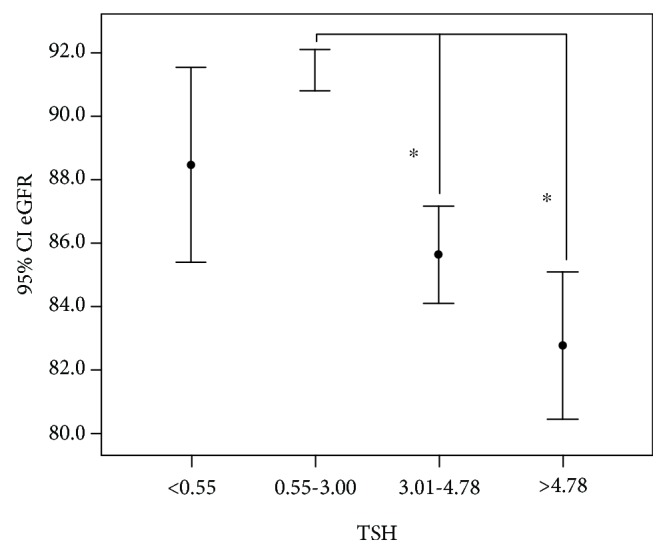
The mean and 95% CI of eGFR in 5936 participants in type 2 diabetes without overt thyroid dysfunction according to different TSH levels. ^∗^*p* < 0.05.

The participants with a lower TSH level between 0.55 and 3.00 had a significant higher eGFR (91.4 ± 22.2 ml/min/1.73 m^2^) than those with a higher TSH level (TSH 3.01–4.78, 85.6 ± 22.7 ml/min/1.73 m^2^, *p* < 0.01), those with subclinical hypothyroidism (82.8 ± 22.4 ml/min/1.73 m^2^, *p* < 0.01), and those with subclinical hyperthyroidism (88.5 ± 24.6 ml/min/1.73 m^2^, *p* < 0.05, Figure 4). The levels of UAE and the prevalence of DN were not significantly different among the four groups (TSH < 0.55 *μ*IU/ml, 0.55 to 3.00 *μ*IU/ml, 3.01 and 4.78 *μ*IU/ml, and >4.78 *μ*IU/ml).

When all the 5936 subjects included in the analysis were further divided into 4 quartiles according to the TSH levels (<1.168, 1.168–1.797, 1.798–2.694, >2.694 *μ*IU/ml), the TSH levels were negatively correlated with the eGFR levels. The participants in the third and the forth quartiles of the TSH group had significant lower eGFR than other groups (92.1 ± 22.9 first quartile, 92.4 ± 21.6 second, 89.3 ± 22.5 third, and 86.0 ± 22.7 forth, all *p* < 0.01). There is no relationship in UAE levels associated with TSH levels.

### 3.5. Correlation between eGFR and TSH

Pearson correlation analysis indicated that there were negative relationships between eGFR and TSH (*r* = −0.086, *p* < 0.01), eGFR and age (*r* = −0.580, *p* < 0.01), eGFR and the duration of diabetes (*r* = −0.270, *p* < 0.01), and eGFR and hypertension (*r* = −0.195, *p* < 0.01). The above correlations were similar in males or females, regardless of hypertension. By contrast, no correlation was found between eGFR and FT_4_.

Linear regression analysis showed that eGFR was significantly negatively associated with TSH (OR: 0.416, 95% CI: 0.321–0.539, *p* < 0.01). The association remained significant after adjustment of age and gender (OR: 0.403, 95% CI: 0.276–0.589, *p* < 0.01) and further adjustment for diabetes duration, HbA1c, TC, TG, and HDL (OR: 0.519, 95% CI: 0.291–0.927, *p* < 0.05). Binary logistic regression showed that TSH is an important risk factor for CKD (OR: 1.036, 95% CI: 1.005–1.068, *p* < 0.05), but the statistical significance disappeared after adjustment for gender and age.

## 4. Discussion

In this cross-sectional population study, high TSH levels were associated with reduced eGFR in type 2 diabetes patients without overt thyroid dysfunction. The association was independent of age, gender, diabetes duration, usage of metformin, HbA1c, TC, TG, and HDL levels.

Results from previous studies suggested that overt and subclinical hypothyroidisms were associated with reduced GFR and higher prevalence of CKD [8–11]. Our results support these observations and, in addition, suggest that low thyroid function within the clinically normal range is associated with reduced GFR and high prevalence of CKD.

Thyroid hormones influence kidney function by direct renal effects as well as systemic hemodynamic [16], metabolic, and cardiovascular effects [17]. Previous studies showed that overt and subclinical hypothyroidisms were associated with reduced eGFR and increased risk of CKD and unresolved subclinical hypothyroidism were independently associated with the rate of renal function decline [12], and thyroid hormone supplement attenuated the alterations [18–20].

It has been suggested that GFR may increase following thyroid hormone supplement in hypothyroidism [18–22], while GFR may be reduced after treatment for hyperthyroidism [21] or after withdrawal of T4 treatment [23]. These findings suggest that low thyroid function may cause reduction in GFR.

Hypothyroidism may decrease ventricular diastolic function [24] and cardiac output, increase peripheral vascular resistance, and result in reduction in renal blood flow [25]. These effects may be ameliorated by thyroid hormone treatment [22, 24]. Subclinical hypothyroidism is also associated with endothelial dysfunction [26] and impaired microvascular function [27]. These findings suggest that low thyroid function might lead to reduced GFR.

On the other hand, it has also been suggested that decreased renal function may influence the thyroid function. The association of different types of glomerulopathies with both hyper- and hypofunction of the thyroid has been reported [28]. Acute kidney injury and chronic kidney disease are accompanied by notable effects on the hypothalamus-pituitary-thyroid axis. The secretion and clearance of pituitary thyrotropin (TSH) is impaired in uremia [28].

Our large study showed that the prevalence of CKD increased with elevated TSH levels, 9.5% (427 out of 4476) in participants with TSH from 0.55 to 3.00 *μ*IU/ml, 13.1% (111 out of 850) in participants with TSH from 3.01 to 4.78 *μ*IU/ml, and 16.0% (58 out of 362) in participants with subclinical hypothyroidism.

Furthermore, a significant inverse association between eGFR and TSH levels was observed throughout whole TSH ranges, which was in accordance with the previous studies [8]. Many situations and medication have effects on kidney functions, such as hypertension [2], lipid profiles [3], glycemic variability [4], and the renin-angiotensin-aldosterone system blockade [5]. Linear regression analysis did not show the association between eGFR and the presence of hypertension or the usage of ACEI/ARB. It might because of the high prevalence of hypertension and the usage of ACEI/ARB in our studied subjects. This relationship was independent of age, gender, duration of diabetes, HbA1c, TC, TG, and HDL levels. Thyroid function, also within the clinically normal range [16], has been positively associated with effective renal plasma flow [22, 28], supporting our results.

Controversies exist as to the reference range for “normal” TSH. Some suggested that TSH upper limit should be decreased from 4.5–5.0 mIU/ml to 2.5–3.0 mIU/ml based on a higher risk of the progression to organ damage [29]. Our evidence supports this view that the prevalence of CKD in participants with TSH from 3.01 to 4.78 *μ*IU/ml was higher than that in participants with TSH from 0.55 to 3.00 *μ*IU/ml and similar to that in participants with subclinical hypothyroidism (TSH > 4.78 *μ*IU/ml). The optimal range of TSH needs further investigation.

Albuminuria is associated with the progression of diabetic kidney disease [30], a faster decline in GFR [31], and an important risk factor of cardiovascular disease [32]. The definition of DN, microalbuminuria, or macroalbuminuria may be inaccurate. These may contribute to the insignificant difference among different TSH groups.

There are some limitations in the present study. First, this is a cross-sectional investigation that demonstrated the association between TSH and CKD, which do not provide information on a causation relationship. Second, we used creatinine-based estimates of GFR. Creatinine production was reduced and tubular secretion was increased in overt hyperthyroidism [33, 34], and therefore, GFR might be overestimated. However, it is not known if creatinine kinetics is influenced by subclinical thyroid dysfunction or thyroid function within the normal range. Smaller studies that have used inulin or ^51^Cr-EDTA clearance to estimate GFR [22, 35, 36] have found that hypothyroidism may be associated with reduced GFR, and these methods do not depend on creatinine levels to estimate GFR. The accuracy of CKD-EPI equation to estimate GFR has already confirmed and validated in the previous studies [37, 38]. Third, the reference range for “normal” TSH was under debate. The relationship between TSH and CKD needs more qualitative analysis. A linear association between TSH and eGFR is supplementary to this limitation. Fourth, the glycemic variability was not measured in the present study. Increasing evidence showed that increased glycemic variability positively associated with chronic complications in diabetic patients [39]. Patients with higher HbA1c had larger glycemic variations [40]. The levels of HbA1c in the present study were moderately higher. The patients under sulphonylureas or insulin therapy, thought to be with worse *β* cell function and longer duration, had larger glycemic variation. Similar portions of patients under sulphonylureas or insulin therapy among three groups might imply that the influence of glycemic variations is limited. Therefore, further large prospective studies were needed to confirm the detrimental effect of high TSH on the kidney.

## 5. Conclusion

High TSH was independently associated with the decreased eGFR in patients with T2D without overt thyroid dysfunction. Our findings indicate that doctors who treat T2D patients should routinely measure the thyroid function.

## Figures and Tables

**Table 1:** Clinical characteristics of the study participants in different thyroid function groups.

	Total (*n* = 5936)	Subclinical hyperthyroidism (*n* = 248)	Euthyroid (*n* = 5326)	Subclinical hypothyroidism (*n* = 362)
Age (years)	62 ± 11^#^	64 ± 11^∗^	62 ± 11	66 ± 10^∗^
Gender (male, %)	2909 (49.0)^#^	133 (53.6)	2670 (50.1)	106 (29.3)^∗^
BMI (kg/m^2^)	24.9 ± 3.5^#^	24.1 ± 3.0^∗^	24.9 ± 3.5	25.4 ± 3.9^∗^
eGFR (ml/min/1.73 m^2^)	89.9 ± 22.6^#^	88.5 ± 24.6	90.5 ± 22.4	82.7 ± 22.4^∗^
UAE (mg/24 h)	90 ± 231	90 ± 198	88 ± 229	114 ± 278^∗^
HbA1c	9.2 ± 2.6^#^	9.6 ± 2.1^∗^	9.2 ± 2.6	8.6 ± 1.9^∗^
Hypertension	3547 (59.8)	144 (58.1)	3167 (59.5)	236 (65.2)^∗^
Duration of diabetes	8.2 ± 0.1^#^	8.7 ± 0.5	8.1 ± 0.1	9.0 ± 0.4^∗^
Duration of hypertension	11.1 ± 8.8	11.8 ± 10.1	11.0 ± 8.7	11.2 ± 8.9
ACEI/ARB (%)	1614 (45.5)	53 (36.8)^∗^	1445 (45.6)	116 (49.2)
Metformin (%)	1127 (19.0)^#^	38 (15.3)	1001 (18.8)	88 (24.3)^∗^
Insulin (%)	1956 (33.0)	86 (34.7)	1759 (33.0)	111 (30.7)
Sulphonylureas (%)	1926 (32.4)	88 (35.5)	1736 (32.6)	102 (28.2)
CKD (%)	629 (10.6)^#^	33 (13.3)	538 (10.1)	58 (16.0)^∗^
Stage				
I	3418 (57.7)	135 (54.4)	3130 (58.8)	153 (42.3)
II	1889 (31.8)	80 (32.3)	1658 (31.1)	151 (41.7)
III	539 (9.1)	26 (10.5)	466 (8.8)	47 (13.0)
IV	71 (1.2)	5 (2.0)	57 (1.1)	9 (2.5)
V	19 (0.3)	2 (0.8)	15 (0.3)	2 (0.6)
Abnormal UAE (%)	2087 (35.2)	98 (39.5)	1847 (34.7)	142 (39.2)
Microalbuminuria (%)	1610 (27.1)	79 (31.9)	1420 (26.7)	111 (30.7)
Macroalbuminuria (%)	477 (8.0)	19 (7.7)	427 (8.0)	31 (8.6)

^∗^ *p* < 0.05, compared with euthyroid group; ^#^*p* < 0.05, compared among subclinical hyperthyroidism, euthyroid, and subclinical hypothyroidism. BMI: body mass index; eGFR: estimated glomerular filtration rate; UAE: 24-hour urine albumin excretion; HbA1c: glycated hemoglobin; ACEI: angiotensin-converting enzyme inhibitors; ARB: angiotensin II receptor blockers; CKD: chronic kidney disease, eGFR < 60 ml/min/1.73 m^2^; stage I, eGFR ≥ 90 ml/min/1.73 m^2^; stage II, 90 > eGFR ≥ 60 ml/min/1.73 m^2^; stage III, 60 > eGFR ≥ 30 ml/min/1.73 m^2^; stage IV, 30 > eGFR ≥ 15 ml/min/1.73 m^2^; stage V, eGFR < 15 ml/min/1.73 m^2^; abnormal UAE, UAE > 30 mg/24 h; microalbuminuria, UAE 30–299 mg/24 h; macroalbuminuria, UAE ≥ 300 mg/24 h.

## Data Availability

The datasets generated and analyzed during the current study are not publicly available but are available from the corresponding author on reasonable request.

## Additional Points

*Highlights*. In patients with T2D, subclinical hypothyroidism was associated with lower eGFR, a higher incidence of CKD, and higher UAE. eGFR was significantly negatively associated with TSH.
